# Comparison between patients with COPD and healthy subjects on spatiotemporal, moment and kinematic parameters: A quasi‐experimental study

**DOI:** 10.1002/hsr2.1784

**Published:** 2024-01-04

**Authors:** Ali Molouki, Mohsen Abedi, Mohammad Mohsen Roostayi, Mobina Khosravi, Mehdi Rezaei

**Affiliations:** ^1^ Department of physiotherapy, School of Rehabilitation Shahid Beheshti University of Medical Sciences Tehran Iran; ^2^ Pulmonary Rehabilitation Research Center (PRRC), National Research Institute of Tuberculosis and Lung Disease (NRITLD) Shahid Beheshti University of Medical Sciences Tehran Iran

**Keywords:** COPD, gait, physiotherapy

## Abstract

**Background and Aims:**

Chronic obstructive respiratory diseases (COPD) not only cause damage to the respiratory system as well as the heart and blood vessels of the patient but also have a direct effect on the condition of the musculoskeletal system. The risk of falling is increasing due to dysfunction of the joints as well as aging, which occurs frequently in this population. Gait deficits are known as an important risk factor for falls. This research aimed to investigate the gait of COPD patients compared to healthy people to gain a better understanding of the reasons for falls.

**Methods:**

Twenty patients with COPD and 20 age and BMI‐matched healthy individuals were included in this study. Sixteen markers were applied to the lower body of the subjects. Spatio‐temporal, kinematic, and maximum moment parameters were measured in different phases in three lower body joints, including the hip, knee, and ankle.

**Results:**

The results showed that all spatio‐temporal parameters in patients were significantly lower than in healthy people. The ankle angle in the sagittal plane at initial contact was significantly difference (*p* = 0.03). As well as, in the frontal plane the hip angle in the mid‐stance showed a significant difference (*p* = 0.02). There was also a significant difference in maximum hip moment in the sagittal plane between the two groups (*p* = 0.01).

**Conclusion:**

The larger hip angle of the patients can be related to the balance problems in the mediolateral direction. The moment showed a significant difference in the hip joint. Since the hip muscles are directly in a synergistic relationship with the trunk muscles, it seems the performance of these muscles is likely to be seriously damaged due to respiratory diseases.

## 1 INTRODUCTION

Chronic obstructive pulmonary disease (COPD) is a *chronic inflammatory lung disease* that causes obstructed airflow from the lungs.^1^ Smoking, air pollution, chemical exposure, severe asthma or bronchitis, and Alpha‐1 antitrypsin deficiency; a genetic disorder can be the causes of this disease.^2^ Although COPD is primarily a lung disease, it is systematically associated with disorders in the musculoskeletal system and motor function of the patients.^3^ Accidental fall is one of the systematic problems related to COPD, which results in decreased quality of life and life expectancy.^3^ Gait deficits, along with balance deficits, impaired activity of daily living, depression and cognitive problems, and medication are known as the main risk factors for falling in these patients.^4^

According to the results of research gait deficits are important risk factors for falling in COPD patients.^5^ The results of a study showed a significant relationship between the severity of the COPD disease and clinical results.^6^ Researchers found that fatigue had a significant effect on the gait variables in patients with COPD.^7^ The results of a study show shortness of breath, physical activity and quality of life reduction in patients with COPD.^8^ Also, another study evaluated gait patterns and the results indicated the deterioration of gait rhythm and disturbance of movement rhythm, as well as a higher risk of falling in patients, which may have happened due to the limitation of airflow to the lungs.^9^

Generally, the effects of COPD on the skeletal‐muscular condition have been neglected to some extent it is referred to as an internal disease and clinically the environmental disorders of gait disorder have not been given enough importance. Biomechanical disorders in COPD patients and their relationship with problems in gait have not been conducted in coherent and comprehensive research, and there are many ambiguous points in the relevant literature in this area. However, the spatio‐temporal, kinematic, and moment parameters in COPD patients are presently not well known comparing healthy people. Studying these parameters can help to reach a better perspective for the therapist regarding the effect of the COPD disease on these people, especially in the issue of falling, which is a common thing among them. Therefore, this study aimed to investigate differences in gait parameters between patients with COPD and healthy individuals on both frontal and sagittal planes.

## 2 MATERIALS AND METHODS

### 2.1 Participants

Forty people, including 20 patients with COPD (study group) and 20 healthy individuals (control group), have participated in the study. Table 1 demonstrates the demographic information of the patients recruited. Subjects were selected for the clinical tests by a lung specialist doctor and introduced to the research team. The main diagnostic components include these two items in the clinical tests: (1) FEV1 (Forced Expiratory Volume in the First Second), the amount of air that leaves the lungs during the first second of forced and high‐pressure exhalation that starts after TLC (Total Lung Capacity). (2) Forced Vital Capacity (FVC), the volume of air that can be expelled from the lungs with maximum force after a deep breath. To diagnose the disease, the FEV1/FVC ratio was evaluated. This ratio decreases in patients with obstructive lung diseases; a ratio of less than 70% indicates the possibility of a person suffering from obstructive lung disease.^10^, ^11^ Thus based on this test, 20 participants were categorized as patients.

**Table 1:** Participants demographics.

	Healthy Mean (SD)	COPD Mean (SD)	*p* Value
Age	57.2 (7.1)	59.3 (3.7)	0.819
BMI (kg/$m^{2}$)	25.1 (1.2)	26.6 (0.5)	0.62
Sex	men	men	‐
Duration of COPD (months)	‐	13	‐
Fall history (times yearly)	0.2	3	‐

Abbreviations: BMI, body mass index; COPD, chronic obstructive respiratory diseases; SD, standard deviation.

The inclusion criteria for COPD patients population were as follow: receiving medical treatment due to respiratory problems, but had not received continuous rehabilitation treatment during the last 2 years, non‐smokers, no need for oxygen capsules during testing or simple daily activities, had no history of central nervous system damage, diabetes, spine, chest, hip, and lower limb joint surgery, and no history of any floor muscle dysfunction. The healthy participants had the same inclusion criteria except for the diagnosis of COPD and receiving medical treatment due to respiratory problems.

Exclusion criteria were pelvis and incontinence, fractures and dislocations in the pelvis and spine, scoliosis and other structural disorders of the spine, chest and organ abnormalities as well as malignancy, rheumatic diseases, systemic and metabolic diseases and vision and vestibular disorders. Also the unwillingness of people to continue cooperation at any stage or the emergence of interfering factors that could change the test result. All the participants signed the consent form to participate in the tests with complete satisfaction. This research has been officially approved by the ethics committee at Shahid Beheshti University of Medical Sciences (IR.SBMU‐RETECH.REC.1398.207).

### 2.2 Sample size

The participants in this research included 40 men, of which 20 were in the COPD group and 20 were in the healthy group. The number of patients according to the presence of two independent groups and being quantitative of dependent variables, assuming *α* = 0.5 and *β* = 0.2 and by examining the data of pilot tests related to six persons was determined.

### 2.3 Measurement tools

To evaluate the parameters, a Vicon motion analysis system (Metrics, UK, 640 Oxford) equipped with eight 120‐Hz infrared cameras synchronized with a Bertec force plate (A, Ohio, Columbus) in Shahid Beheshti University of Medical Sciences was used. Sixteen markers with a diameter of 14 mm were bilaterally placed on the anterior superior iliac spine, the posterior superior iliac spine, and the head of the fifth metatarsus, the first metatarsus, calcaneus, lateral malleolus, and inside and outside the knee joint.

In the first step, the person was asked to walk around the laboratory environment for 5 min and familiarize himself with the laboratory environment. Then, the participants started walking and their markers data were recorded, including their location in the three‐dimensional space of the laboratory. When the foot was placed on the force plate, the force and moment data were recorded. Participants stood at one end of the space of approximately 8 meters of the laboratory and began to move by the vocal clue of the experimenter. In the next step, the foot was placed completely outside the force plane. All tests were repeated three times and between each test, a wash‐out time of at least 5 min was considered.

### 2.4 Gait data analysis

To analyze the gait data, In the Vicon Nexus system 2.12, marker trajectories were labeled and gaps in the trajectories were filled with a maximum gap fill option. After calibration through the dynamic and static pipeline, the data were exported to MATLAB software (2020a, The Math Works, Inc.). The obtained results included: spatio‐temporal characteristics of the gait including walking speed, cadence, stride length, and the number of steps per min. In addition to the angles of the ankle, knee and hip joints in 3‐points of the stance phase, that is, the initial contact, the mid‐stance and the terminal stance. The third series of reported data was the maximum moment of these three joints in the sagittal plane. For the hip joint, the maximum moment in the frontal plane was also presented.

### 2.5 Statistical analysis

The Kolmogorov−Smirnov (K−S) test was utilized to check the data normality. Two‐tailed Independent *t*‐test was used to compare two groups for gait test variables. The significance level was set to 0.05. The statistical analyses were performed in SPSS 20 (IBM Corp, and Armonk).

## 3 RESULTS

The statistical results of spatio‐temporal, kinematics and moment variables are listed in Table 2.

**Table 2:** The mean (SD) and 95% confidence intervals of the statistical analysis to compare the gait variables in two groups of COPD patients and healthy.

	Mean (SD)		95% Confidence interval		*p* Value
	COPD	Healthy	COPD	Healthy	
Velocity (m/s)	1.04 (0.17)	1.11 (0.21)	(−0.95 to 3.03)	(−0.89 to 3.11)	<0.01*
Cadence (step/min)	98.6 (10.15)	102.2 (12.3)	(102.8−94.37)	(97.49−106.91)	<0.01*
Step length (m)	0.58 (0.1)	0.66 (0.13)	(−2.27 to 2.56)	(−1.32 to 2.64)	<0.01*
Step wide (m)	0.083 (0.03)	0.101 (0.04)	(−1.87 to 2.04)	(−2.02 to 2.23)	<0.01*
Ankle angle in the sagittal plane at initial contact (°)	1 (0. 2)	4 (0.9)	(−1 to 3)	(2.16−6.16)	0.03*
Knee angle in the sagittal plane at initial contact (°)	2 (0.7)	−2 (0.5)	(011−4.11)	(−6.18 to 2.18)	0.23
Hip angle in the sagittal plane at initial contact (°)	37 (6.7)	34 (5.9)	(33.55−40.45)	(30.73−37.27)	0.46
Hip angle in the frontal plane at initial contact (°)	8 (1.1)	3 (0.9)	(6.2−10.2)	(0.84−5.16)	0.13
Ankle angle in the sagittal plane in the mid‐stance (°)	5 (1.4)	8.5 (1.9)	(2.73−7.27)	(6.12−10.88)	<0.01*
Knee angle in the sagittal plane in the mid‐stance (°)	10.5 (4.3)	9 (3.4)	(7.58−13.42)	(6.28−11.72)	0.33
Hip angle in the sagittal plane in the mid‐stance (°)	18 (9.2)	13 (5.2)	(13.99−22.01)	(10.12−16.12)	0.02*
Hip angle in the frontal plane in the mid‐stance (°)	−7 (2.7)	−3 (1.6)	(−9.56 to 5.56)	(−5.31 to 1.31)	0.5
Ankle angle in the sagittal plane at the terminal stance (°)	−6 (2. 2)	−7 (1.9)	(−1 to 3)	(2.16−6.16)	0.24
Knee angle in the sagittal plane at the terminal stance (°)	35 (7)	30 (6.6)	(0.11−4.11)	(−4.07 to 0.7)	0.09
Hip angle in the sagittal plane at the terminal stance (°)	3 (1.1)	−3 (0.7)	(1.02−5.02)	(−5.11 to 1.11)	0.19
Hip angle in the frontal plane at the terminal stance (°)	4 (1.9)	9 (2.4)	(2.38−6.38)	(7.49−11.49)	0.04*
Ankle moment in the sagittal plane in the mid‐stance (N × m/kg)	1.2 (0.46)	1.3 (0.11)	(−0.86 to 3.26)	(−0.68 to 3.28)	0.11
Knee moment in the sagittal plane in the mid‐stance (N × m/kg)	0.72 (0.35)	0.61 (0.31)	(−1.33 to 2.77)	(−1.41 to 2.63)	0.21
Hip moment in the sagittal plane in the mid‐stance (N × m/kg)	0.86 (0.11)	0.74 (0.15)	(−1.12 to 2.84)	(−1.25 to 2.73)	<0.01*
Hip moment in the frontal plane in the mid‐stance (N × m/kg)	0.52 (0.15)	0.59 (0.23)	(−1.47 to 2.51)	(−1.42 to 2.6)	0.17

Abbreviations: COPD, Chronic obstructive respiratory diseases; SD, standard deviation.

* Significant at *α* level of 0.05.

The results showed that the spatiotemporal variables of the gait, including stride length stride width, speed and cadence in COPD, were all significantly lower than in healthy people (*p* < 0.05) so the speed decreased from 1.11 to 1.04 m/s. Also, the cadence decreased from 102.2 to 98.6 step/min in patients compared to the control group. Stride characteristics including length and width also showed a decrease from 66 to 58 cm and also from approximately 10 to 8 cm in COPD patients.

Results of the lower body joint angles, including the hip, knee and ankle, at three points of the individual's gait cycle at the moment of initial contact, the mid‐stance and the terminal stance, were reported in Table 2. The angle of the ankle in the sagittal plane in the initial contact and the mid‐stance in COPD patients was significantly lower than in healthy individuals (*p* = 0.01). Furthermore, Table 2 indicated that the ankle maximum moment was significantly lower in COPD patients (*p* = 0.11). On the contrary, the hip angle in the sagittal plane in these patients at the mid‐stance was significantly larger than healthy subjects (18° vs. 13°), at the moment of initial contact, this value was also higher, but it was not significant (*p* = 0.13). The hip adduction angle of COPD patients was also significantly lower than the control group at the terminal stance phase (4° vs. 9°). It can be understood that the hip moment in the sagittal plane is significantly smaller in healthy people than in patients, although the moment in the frontal plane is greater for healthy people, however, this difference was not significant (*p* = 0.17). No significant difference was found for the knee angle in the stance. The COPD knee angle at the terminal stance was 5° larger than the control group however, it was not significant (*p* = 0.09).

**Figure 1:**
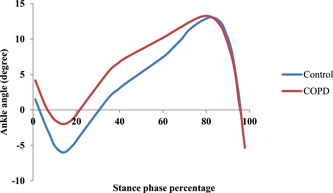
Changes in ankle angle during the stance phase for one subject from both groups.

**Figure 2:**
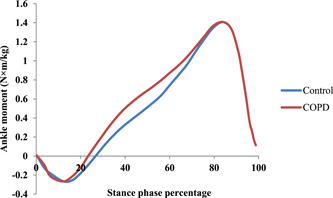
Ankle moment changes during the stance phase for one subject from both groups.

In addition, the time patterns of the ankle angle and ankle moment were reported in Figures 1 and 2, respectively. This pattern was shown from zero to 100% of the stationary phase of the entire gait cycle. As it is clear from Figure 1, the difference in the ankle angle in both healthy and patient groups is more at the beginning and mid‐stance and they are almost similar when reaching the maximum angle. The moment at the mid‐stance shows the biggest difference between the two groups (Figure 2).

## 4 DISCUSSION

This study aimed to explore the differences in gait parameters between patients with COPD and healthy individuals on both frontal and sagittal planes. Our findings indicated lower spatio‐temporal and kinematic parameters in COPD patients. The angle of the ankle in both mid‐stance and initial contact was significant in the patient's group. Also, more hip angle was reported in the patient's group.

Gait deficits in COPD patients such as reduced cadence and stride length have been confirmed in several studies.^12^, ^13^, ^14^ Also, in other studies that used the 6‐min walk test as a clinical test, it has been determined that the movement speed of these patients was lower than healthy individuals who are in the same age range and body mass.^15^, ^16^ In one investigation, Laos et al. concluded that the speed and cadence are directly related to the FEV1/FEC percentage; limitation in the intake air and exhalation frequency, and the problem of distinguishing COPD patients is based on their gait characteristics.^9^ Another issue that has affected the cadence and stride length of these people is the increase in walking time in these patients.^17^ These people walked a shorter distance than the control group in the 6‐min walking test. The reason for the increase in walking time can be justified by the history of falling in these people, which is a strategy in these patients so that they can increase their stability while walking and prevent falling.^13^, ^14^ In another study between COPD patients who had a history of falling and individuals who had a history of falling without COPD, it was shown that people with COPD had a significantly lower speed and cadence than the other group.^9^ The research conducted on the kinematic and kinetic characteristics of COPD gait indicated that in the mid‐stance, the angle and moment of the ankle in these patients is less than the control group under fatigue conditions,^7^ which is probably caused by the problem in the function of the Tibialis anterior muscle, which has been subjected to long‐term pressure due to lack of required oxygen and musculoskeletal deformities in the anterior‐posterior direction, such as kyphosis of the spine.^18^, ^19^ In addition, the problem of lack of oxygen due to abnormal breathing in these people is probably an important factor in reducing the speed and abnormality of walking in these patients.^9^ The gait of COPD patients was investigated by Yents et al. under fatigue, so after walking for a few minutes, their results were analyzed, and it was found that the stride length of these patients under fatigue was greater than healthy participants under fatigue, which is probably because the Tibialis anterior muscle has been able to adapt to this problem over time due to the lack of oxygen, and it has maintained the trend by requiring less oxygen under fatigue. But in healthy people, when a person feels tired, he has the same natural reaction and the steps become smaller.^7^ Another important thing that was obtained from another similar research is that the step width has decreased in COPD people in a state of fatigue, but the step width has increased in healthy people. The step width variable is directly related to the risk of falling so its small value is related to the smaller base of support.^20^, ^21^

This problem is interpreted as an increase in instability in the mediolateral direction, so that the center of pressure is outside the balance range and the person becomes prone to falling,^7^, ^22^ thus increasing the length of the step and decreasing the width of the step. In these patients, it helps to understand the behavior of these patients when they are exhausted when their breathing problem is more visible and maintain balance during gait, which probably increases the stride length by increasing the hip angle in the sagittal plane.^23^ However, due to muscle weakness and problems in the medial‐lateral direction, they could not prevent the reduction of their stride width, and problems in their balance will arise on the balance on the lateral side, although it has caused the differences in the gait indices between them and healthy people to decrease, but the risk of falling increases, which shows the weakness of the motor control in these people. The current research has also reported an increase in the hip angle in the sagittal plane and a decrease in this angle in the frontal plane.

The shortness of breath caused by these people due to the double effort to draw air in while not completely emptying the exhaled air from the lungs, over time has caused the functional weakness of these people which decreases during the review of their daily activities for a few minutes in the day.^4^ COPD causes problems in the function and structure of muscles.^24^ Complications such as the destruction of muscle cells, atrophy and reduction of mitochondrial density, as well as the change of muscle fiber from type one to type two, which can reduce muscle oxygenation, are common in these patients.^25^ This change of nature to type two muscles can be a major reason for increasing the possibility of muscle fatigue and weakness during physical activities.^26^ Weakness of quadriceps muscles and ankle dorsi flexor and plantar flexor muscles have been reported in COPD patients,^27^, ^28^, ^29^ as well as postural deformities and slow reaction time to disturbances. The environment has increased the risk of falling and, as a result, problems such as bone fracture in these patients.^4^ It seems that the problems of these patients in the direction of mediolateral can be related to the fluctuations of the trunk due to muscle weakness and lack of proper postural control during inhalation and exhalation.^23^ The deficits in the ankle muscles have significantly affected gait and postural balance in patients with COPD, especially in the direction of anterior‐posterior. In addition, the condition of the trunk muscles in a synergistic connection with the weakness of their quadriceps muscles has created balance problems in the direction of their mediolateral. Imbalance in the walking of these people, as their most common daily activity, can be the cause of falls and severe injuries in these people. Gait analysis can clarify problems and details that cannot be detected normally. For example, the moment characteristics of the joints can help to estimate the net effect of the muscles on the joint without having their activity data and provide the right way to treat the person; especially the therapist can prepare the right strategies to prevent the patient from falling. Some limitations need to be noted regarding the present study. The present study evaluated a limited age range (50−65 years) in men. Therefore, the results may not be generalizable to all age groups. Further research is required in more age ranges and under different test conditions, such as stepping up or down with new analysis methods such as IMU sensors.^30^ Moreover, another limitation of this study was the lack of assessment of muscle strength, a domain suggested for examination in future investigation.

## 5 CONCLUSION

This research, focusing on the analysis of the gait of COPD patients, showed that the spatio‐temporal characteristics of the gait of these patients are significantly lower than those of healthy people. Probably, the finding about the COPD‐related decrease in step widths indicates an unstable walking in the mediolateral direction and a reason for frequent falling in these people. Furthermore, gait problems found in COPD's ankle kinematics would be related to the weakness of the Tibialis Anterior muscle, and strengthening this muscle might be important to improve their gait. Also, increasing the hip angle in the sagittal plane and decreasing it in the frontal plane, which is synergistically connected with the weakness of the trunk muscles, is effective in the formation of instability in these people.

## AUTHOR CONTRIBUTIONS

**Ali Molouki**: Formal analysis; investigation; methodology; writing—original draft. **Mohsen Abedi**: Funding acquisition; project administration; supervision. **Mohammad Mohsen Roostayi**: Supervision; writing—review & editing. **Mobina Khosravi**: Resources; writing—original draft. **Mehdi Rezaei**: Data curation; investigation.

## CONFLICT OF INTEREST STATEMENT

The authors declare no conflict of interest.

## TRANSPARENCY STATEMENT

The lead author Mohsen Abedi affirms that this manuscript is an honest, accurate, and transparent account of the study being reported; that no important aspects of the study have been omitted; and that any discrepancies from the study as planned (and, if relevant, registered) have been explained.

## Data Availability

The data that support the findings of this study are available from the corresponding author, upon reasonable request.
